# Luteinizing hormone induces expression of 11beta-hydroxysteroid dehydrogenase type 2 in rat Leydig cells

**DOI:** 10.1186/1477-7827-7-39

**Published:** 2009-05-04

**Authors:** Qian Wang, Ping Zhang, Hui-Bao Gao

**Affiliations:** 1 Department of Biochemistry and Molecular Biology, Shanghai Jiao Tong University School of Medicine, 280 South Chong Qing Road, Shanghai 200025, PR China; 2 Institutes of Medical Sciences, Shanghai Jiao Tong University School of Medicine, 280 South Chong Qing Road, Shanghai 200025, PR China

## Abstract

**Background:**

Leydig cells are the primary source of testosterone in male vertebrates. The biosynthesis of testosterone in Leydig cells is strictly dependent on luteinizing hormone (LH). On the other hand, it can be directly inhibited by excessive glucocorticoid (Corticosterone, CORT, in rats) which is beyond the protective capability of 11beta-Hydroxysteroid dehydrogenase type 1 (11beta-HSD1) and type 2 (11beta-HSD2; encoded by gene Hsd11b2 in rats) in Leydig cells. Our previous study found that LH increases 11beta-HSD1 expression in rat Leydig cells, but the effect of LH on the expression and activity of 11beta-HSD2 is not investigated yet.

**Methods:**

The Leydig cells were isolated from male Sprague-Dawley rats (90 days of age). After Leydig cells were incubated either for 24 h with various concentrations of LH (2.5, 5, 10 and 20 ng/mL) or for different time periods (2, 8, 12 and 24 h) with 20 ng/mL LH, the mRNA expression of 11beta-HSD2 was measured by real-time PCR. 11beta-HSD2 protein levels in Leydig cells were assayed by Western Blot and 11beta-HSD2 enzyme activity was determined by calculating the ratio of conversion of [3H]CORT to [3H]11-dehydrocorticosterone by 24 h after stimulation with 20 ng/ml LH. Four reporter gene plasmids containing various lengths of Hsd11b2 promoter region were constructed and transfected into mouse Leydig tumor cells to investigate the effect of LH on Hsd11b2 transcription. A glucocorticoid-responsive reporter gene plasmid, GRE-Luc, was constructed. To evaluate influence of LH on intracellular glucocorticoid level, rat Leydig cells were transfected with GRE-Luc, and luciferase activities were measured after incubation with CORT alone or CORT plus LH.

**Results:**

We observed dose- and temporal-dependent induction of rat 11beta-HSD2 mRNA expression in Leydig cells subject to LH stimulation. The protein and enzyme activity of 11beta-HSD2 and the luciferase activity of reporter gene driven by promoter regions of Hsd11b2 were increased by LH treatment. LH decreased the glucocorticoid-induced luciferase activity of GRE-Luc reporter gene.

**Conclusion:**

The results of the present study suggest that LH increases the expression and enzyme activity of 11beta-HSD2, and therefore enhances capacity for oxidative inactivation of glucocorticoid in rat Leydig cells in vitro.

## Background

In the male, the Leydig cell, in the interstitium of testis, is the primary source of sexual steroid hormone testosterone, which stimulates differentiation of the male phenotype and spermatogenesis in the testes. Leydig cells are mainly stimulated by luteinizing hormone (LH), the gonadotropic hormone secreted by pituitary gland. On the other hand, many studies had established that the high level of glucocorticoid, which could be caused pathologically by Cushing's syndrome or psychologically by stress, results in the decrease in testosterone secretion, whereby reduced libido and fertility are brought on [1,2]. Furthermore, our previous study found that the endogenous glucocorticoid (Corticosterone, CORT, in rats) at physiological level is also able to suppress the secretion of testosterone in rat Leydig cells [3]. In our another study, it was shown that administration of stress level of glucocorticoid induces apoptosis of Leydig cells *in vivo *[4]. It suggests that glucocorticoid could decrease testosterone production by Leydig cells through reducing the number of Leydig cells as well as inhibiting the expression of testosterone biosynthesis enzymes [5-8]. The adverse effect of glucocorticoid on testosterone biosynthesis is a direct glucocorticoid receptor (GR) mediated process [9]. The intracellular concentration of glucocorticoid, which determines the extent of GR activation, is regulated by 11beta-hydroxysteroid dehydrogenase (11β-HSD) in Leydig cells [10]. To date, two isoforms of 11β-HSD are identified. 11β-HSD Type I (11β-HSD1) is a NADP^+^/NADPH dependent oxidoreductase with low affinity (km = 2 μM) for glucocorticoid, reversibly converting biologically active glucocorticoid (corticosterone in rats) to inactive 11-keto steroid [11]. Its direction of enzyme activity is determined by redox potential in different cell types and differentiation stages [12]. In rat Leydig cells, 11β-HSD1 is a predominant oxidase, playing a protective role in the inhibitory effect of glucocorticoid on steroid biosynthesis of Leydig cells [13].11β-HSD Type II (11β-HSD2; encoded by rat gene *Hsd11b2*) is a NAD^+ ^dependent oxidase with high-affinity (Km = 15 nM), inactivating glucocorticoid to its inert metabolite (11-dehydrocorticosterone in rats) unidirectionally [14]. Both of two isoforms of 11β-HSD are expressed in rat Leydig cells [15,16]. Although the expression of 11β-HSD2 is 1000-fold lower relative to 11β-HSD1 in rat Leydig cells, the former is still thought of as an important factor, at least equivalent to 11β-HSD1, of modulating the intracellular level of glucocorticoid due to its high affinity for substrate [15]. In a word, the excessive glucocorticoid directly inhibits testosterone biosynthesis when it exceeds the capacity of oxidative inactivation by 11β-HSD in Leydig cells. In light of the important role of 11β-HSD in modulating glucocorticoid-mediated suppression of testosterone secretion, our previous study had been performed to investigate whether the expression and activity of 11β-HSD1 is regulated by LH, the primary trophic and stimulating hormone for Leydig cells [17].

The results showed that although LH increases the expression of 11β-HSD1 mRNA and protein compared with control cells, the net oxidative activity of 11β-HSD1 was reduced. A study had shown that gonadotropic hormone which can be used as a functional homologue of LH in teleosts [18] could increase expression of 11β-HSD2 in Tilapia testis [19], but whether LH could regulates its expression in mammalian Leydig cells and whereby affects the intracellular concentration of glucocorticoids is unknown. The present study is designed to investigate the effect of LH on expression of 11β-HSD2 in rat Leydig cells.

## Methods

### Chemicals and animals

Corticosterone (C2505), Luteinizing hormone (L5259), bovine lipoprotein (L3626), Percoll (P1644) and DMEM-Ham's F12 (D2906) were purchased from Sigma-Aldrich Chemical (St. Louis, Missouri, USA). The antibodies for 11β-HSD2 (sc-20176) and β-actin (sc-130657) were obtained from Santa Cruz Biotechnology (Santa Cruz, California, USA). The Dual-Luciferase Reporter Assay System (E1910) was purchased from Promega Co. (Madison, Wisconsin, USA). [^3^H]CORT was kindly provided by Dr. Ren-Shan Ge (Population Council, New York, USA). The Male Sprague-Dawley rats (90 days of age) were purchased from the Animal Centre of the Chinese Academy of Sciences (Shanghai, China). The animals were killed by CO_2 _asphyxiation for isolation of Leydig cells. Mouse Leydig tumor cell line mLTC-1 (CRL-2065) and rat Leydig tumor cell line R2C (CCL-97) were purchased from American Type Culture Collection (Manassas, Virginia, USA).

### Cell isolation and culture

Adult Leydig cells were isolated from 90-day-old rats according to the procedure of Sriraman et al. [20], which is a modification of the procedure described by Klinefelter et al. [21]. The decapsulated testis was subjected to collagenase digestion in a 50-mL plastic tube containing 10 mL medium with collagenase (600 units) and DNase (750 units). The tubes were placed in a shaking water bath with constant agitation (50 times/min) at 34°C for 15–20 min until the seminiferous tubules were separated. The enzyme action was terminated by adding excess medium. The tubules were allowed to settle by gravity and the medium, consisting of interstitial cells, was aspirated and filtered through a 100-μm nylon mesh. The filtrate was centrifuged at 250 × g for 10 min at 25°C, which yielded a crude interstitial pellet. The pellet obtained was suspended in 35 mL 55% isotonic Percoll with 750 units DNase in Oakridge tubes. The tubes were centrifuged at 20 000 × g for 1 h at 4°C. Percoll fractions corresponding to densities of 1.070–1.090 g/mL were collected and the cells present in this fraction were pelleted by centrifugation at 250 × g for 10 min at 25°C after diluting with 3–4 volumes of medium. The purities of isolated cell fractions were evaluated by histochemical staining for 3β-hydroxysteroid dehydrogenase activity, with 0.4 nmol/L etiocholanolone as the steroid substrate [22]. The mean purity of Leydig cells was 85%.

Rat Leydig cells culture was conducted as previously described [23]. Briefly, freshly isolated Leydig cells were seeded in 6-well plates, at a density of 4 × 10^5 ^cells per well, and cultured for 24 h in phenol red-free DMEM/Ham's F12 medium supplemented with 1 mg/mL bovine lipoprotein in an incubator gassed with 5% O_2_, 5% CO_2_, at 34°C.

The mLTC-1 cells were cultured in DMEM medium (Invitrogen) supplemented with 10% fetal bovine serum (FBS); R2C cells were cultured in Ham's F10 medium supplemented with 15% horse serum, 2.5% FBS. Both cell lines were incubated at 37°C in a humidified atmosphere of 95% air and 5% CO_2_.

### RNA isolation and real-time PCR quantitation

Total RNA was extracted from cultured cells using an RNeasy Mini Kit (Qiagen, Valencia, California, USA) coupled with on-column DNase digestion with the RNase-Free DNase Set (Qiagen) according to the manufacturer's instructions. First strand cDNA synthesis was performed using Superscript II reverse transcriptase (Invitrogen, Shanghai, China) in a reaction using 2 μg of total RNA primed with random hexamers in a total reaction volume of 20 μL.

Real-time PCR was carried out according to Lee JJ and Widmaier EP with some modification [24]. In brief, each PCR reaction contains 1 × Absolute TM QPCR SYBR Green Mix (Abgene, Epsom, UK), 0.3 μM primers and 2 μL cDNA with a total volume of 25 μL. The primers for 11β-HSD2 [GenBanK:NM_017081] (Forward: 5'-TGGCCAACTTGCCTAGAGAG; Reverse: 5'-TTCAGGAATTGCCCATGC) and 18S [GenBank:X03205] (Forward: 5'-CGCCGCTAGAGGTGAAATTC; Reverse: 5'-CCAGTCGGCATCGTTTATGG) are the same as described previously [24]. Initially, the reaction mix was incubated at 95°C for 15 min to activate Thermo-Start^® ^DNA Polymerase (Abgene), then templates were amplified for 40 cycles (95°C for 15 s, 60°C for 1 min) on the Rotor-Gene 3000 system (Corbett Research, Sydney, NSW, Australia). Meanwhile, genomic DNA contamination was evaluated by real-time PCR of non-retrotranscribed RNA for each sample. Melting curve analysis was conducted immediately after completion of PCR as following: 95°C for 10 s, then 60°C for 10 s and continuous heating to 99°C at 0.5°C/s. The resulting PCR products were analyzed on a 2% agarose gel and cloned into pGEM-T Easy vector (Promega) followed by DNA sequencing to confirm the specificity of PCR. The relative quantitative analysis of 11β-HSD2 mRNA expression was performed using the delta-delta Ct method with 18S RNA as an internal control [25].

### cDNA cloning of rat 11β-HSD2

Total RNA was prepared from rat kidney tissue using the Trizol Reagent (Invitrogen) according to the manufacturer's instruction. First strand cDNA synthesis was performed using Superscript II reverse transcriptase (Invitrogen) in a reaction using 2 μg of total RNA primed with random hexamers in a total reaction volume of 20 μl. For amplification of rat 11β-HSD2 [GenBanK:NM_017081], the forward and reverse primers were 5'-GAC GGT ACC CGA GTA TCC CTC CCA C-3' and 5'-ACG CTC GAG TCT CCT GCT GAA ACA CCT A-3', respectively. PCR was performed for 30 cycles with denaturing at 94°C for 30 s, annealing at 58°C for 30 s and extension at 72°C for 90 s. The 11β-HSD2 cDNA was purified by QIAquick PCR Purification Kit.

### Plasmid construction

Rat genomic DNA was isolated from tail tissue using DNAeasy kit (Qiagen). Four DNA fragments corresponding to different lengths of rat gene *Hsd11b2 *promoter region [GenBank:NC_005118] were generated using their respective upstream primers and the common downstream primer 5'-cccaagcttagtgcagaggaacaccagcctg-3' in PCR using platinum Pfx high fidelity polymerase (Invitrogen) and 50 ng of genomic DNA template. The upstream primers, 5'-cggggtaccggaatcaatgggtttagaaaag-3', 5'-cggggtaccagcaagagaccttgatgtctg-3', 5'-cggggtaccggcggggcgggggggcacctgc-3' and 5'-cggggtaccggcgctttataagctgggtcc-3' correspond to constructs -1740/+45,-756/+45,-138/+45 and -35/+45, respectively (+1 designates the transcription start site). The resultant fragments were gel-purified, cloned into pGEM-T Easy vector (Promega) and sequenced for fidelity. The four inserts were then subcloned into pGL3-Basic luciferase vector (Promega) after digestion with Kpn I and Hind II.

11β-HSD2 cDNA sequences were digested with Kpn I and Xho II, and ligated into the Kpn I/Xho I site of pcDNA3.0 plasmids (Invitrogen) to construct the 11β-HSD2 expression vector, named pcDNA-hsd11b2.

A luciferase reporter gene plasmid, GRE-Luc was constructed as described previously [26], which transcriptional activity is subject to intracellular glucocorticoid level. Briefly, An oligonucleotide (5'-tatataacgcgttgtacaggatgttctctctgcctctgctgtacaggatgttctagatctgccctatagtgagtcgtattac-3'), which contains two copies of GRE (glucocorticoid response element) consensus sequence (shown underlined), was synthesized. This oligonucleotide was primed with a short oligonucleotide primer and made double-stranded with Klenow enzyme and dNTPs (New England Biolads, Beverly, Massachusetts, USA). After digestion with Mlu I and Bgl II, the DNA was cloned upstream of the simian virus 40 promoter in the pGL3-promoter vector (Promega) to yield the GRE-Luc construct. Similar constructs have been used in other studies to measure responsiveness to glucocorticoids [27,28].

### Overexpression of 11β-HSD2 in R2C cells

R2C cells were seeded in 24 well culture plates (5 × 10^5^/well) in fresh medium for 24 h. Then, 1.0 μg of pcDNA-hsd11b2 was transiently transfected into R2C cells using Lipofectamine™ 2000 reagent (Invitrogen) according to the supplier's protocol. After another 48 h of culture, the expression of 11β-HSD2 protein was identified by Western blotting. R2C cells transfected with pcDNA3.0 vector served as control.

### Western blot analysis

Primary rat Leydig cells and transfected R2C cells were lysed in Ripa buffer (1% NP-40, 0.1% SDS, 0.5% DOC, 150 mM NaCl, 10 mM Tris-HCl, and PMSF mixture) at 4°C for 30 min. Protein concentrations were determined BCA protein assay reagent (Pierce Chemical, Rockford, Illinois, USA). Aliquots of cell extracts containing equal amounts of protein were separated by SDS-polyacrylamide gel electrophoresis on 12% gels using the Laemmli buffering system, then proteins were transferred to ImmunoBlot polyvinylidene difluoride membranes (Bio-Rad, Hercules, California, USA) and blocked by rocking for 1 h at room temperature in blocking buffer (Tris-buffered saline with 0.1% Tween 20 and 5% nonfat dry milk). Afterwards, the membranes were incubated with a 1:1000 dilution of a rabbit polyclonal antibody for 11β-HSD2 for 1 h, then repeatedly washed and incubated with a 1:10000 dilution of goat anti-rabbit antibody that was conjugated to horseradish peroxidase. Signals were detected with an Enhanced Chemilluminescence kit (Pierce Chemical). Beta-actin was used as internal control.

### Measurement of 11β-HSD2 enzyme activity

After rat Leydig cells were isolated and cultured for 24 h, cells were treated with 20 ng/mL LH for 24 h. Then, Leydig cell homogenates were prepared with the method described by Arcuri F et al [29]. In short, cell cultures were rinsed three times with 0.1 M phosphate buffer solution, harvested with a rubber scraper and pelleted by centrifugation at 1000 × g for 5 min at 4°C. Cell pellets were suspended in ice-cold sodium phosphate buffer 0.1 M (pH 7.5), 0.2% (v/v) of Triton X-100, and sonicated for 15 s on ice. Protein concentrations were determined by BCA protein assay reagent (Pierce Chemical). The 11β-HSD2 enzyme activity assay was performed according to the protocol of Ge RS *et al*. with some modification [16]. Briefly, 0.5 mL reaction mixture was prepared in phenol red-free medium that contained 2 nM [^3^H]CORT and 23 nM unlabeled CORT, obtaining a final concentration of 25 nM of CORT. The reactions were initiated by the addition of 50 μg protein with NAD^+ ^at a final concentration of 0.5 mM, then performed in a shaking water bath (100 strokes/min) at 34°C for 1 h, and finally stopped by adding 2 ml ice-cold ethyl acetate. Tubes were vortexed and centrifuged at 10,000 rpm for 5 min to separate the upper organic layer, which was dried under nitrogen. Samples were resuspended in 100 μl of methanol. The steroids were separated chromatographically on thin-layer plates in chloroform and methanol (90:10). Bands were visualized using I_2 _vapor and scraped into scintillation fluid, and the radioactivity was measured using a Beckman LS65000 liquid scintillation counter (Beckman Coulter, Fullerton, USA). The percentage conversion of corticosterone to 11-dehydrocorticosterone was calculated by dividing the radioactive counts identified as 11-dehydrocorticosterone by the total counts associated with corticosterone plus 11-dehydrocorticosterone.

### Transient transfection and reporter gene assay

To determine the effect of LH on transcription of 11β-HSD2 gene, four luciferase reporter genes driven by different lengths of 11β-HSD2 promoter fragment were transiently transfected into mLTC-1. Briefly, the cells were seeded into 24-well plates at a density of 5 × 10^5 ^cells/well and incubated at 37°C overnight. Cells were then placed in DMEM with 10% fetal bovine serum containing 1 μg of luciferase plasmid, 0.01 μg of pRL-TK (Promega) reporter plasmid, and 2 μg of Lipofectamine™ 2000 reagent (Invitrogen) according to the supplier's protocol. Twelve hours following transfection, the cells were treated with 20 ng/ml LH for 24 h. Following the treatment, Dual Luciferase Assay was performed by lysing the cells in Passive Lysis Buffer (Promega) and reading the relative light units of one-fifth the lysate with both the firefly substrate and the renilla substrate using a luminometer (Berthold Lumat LB9507, Bad Wildbad, Germany). Each transfection was performed in triplicate and in three independent experiments.

Given that both isoforms of 11β-HSD are expressed in rat Leydig cell and the inhibitory effect of LH on the net oxidative activity of 11β-HSD1 [17], a glucocorticoid-responsive reporter gene, GRE-Luc, was employed to investigate the level of active intracellular glucocorticoid. The rationale is that the transcriptional activity of GRE-Luc which contains two copies of a consensus GRE upstream of the luciferase gene is subject to the extent of GR activation, i.e. intracellular glucocorticoid concentration set by the total oxidative activity of 11β-HSD1 and 11β-HSD2, thus the effect of LH on the transcriptional activity of GRE-Luc is indicative of the oxidative capacity of Leydig cells for glucocorticoid. Rat Leydig cells were isolated and cultured in 24 well culture plates (1 × 10^6^/well) in fresh medium for 48 h. Then, 1.0 μg of GRE-Luc and 0.01 μg of pRL-TK (Promega) reporter plasmid were transiently transfected into the Leydig cells using Lipofectamine™ 2000 reagent (Invitrogen). After another 48 h of culture, the cells were incubated in the presence or absence of LH (20 ng/mL) for 24 h. Subsequently, the cells pretreated with LH were treated with CORT (50 nM) plus LH (20 ng/mL) and the other cells not pretreated with LH were treated with CORT (50 nM) or LH (20 ng/mL) for 24 h. At the end of all treatments, Dual Luciferase Assays were performed as described above. The transfected cells treated with vehicles (PBS for LH; DMSO for CORT) served as controls.

### Statistics

Results are presented as means ± SE of three independent experiments. Statistical analyses of 11β-HSD2 mRNA data and Luciferase assay data were performed using one-way ANOVA followed by Student-Newman-Kuels test. 11β-HSD2 protein data was analyzed by a standard Student's t-test. Significance was set at *P *< 0.05. Calculations were performed using SPSS software version 10.0 (Chicago, USA).

## Results

### Effect of LH on expression of 11β-HSD2 mRNA and protein in rat Leydig cells

**Figure 1:**
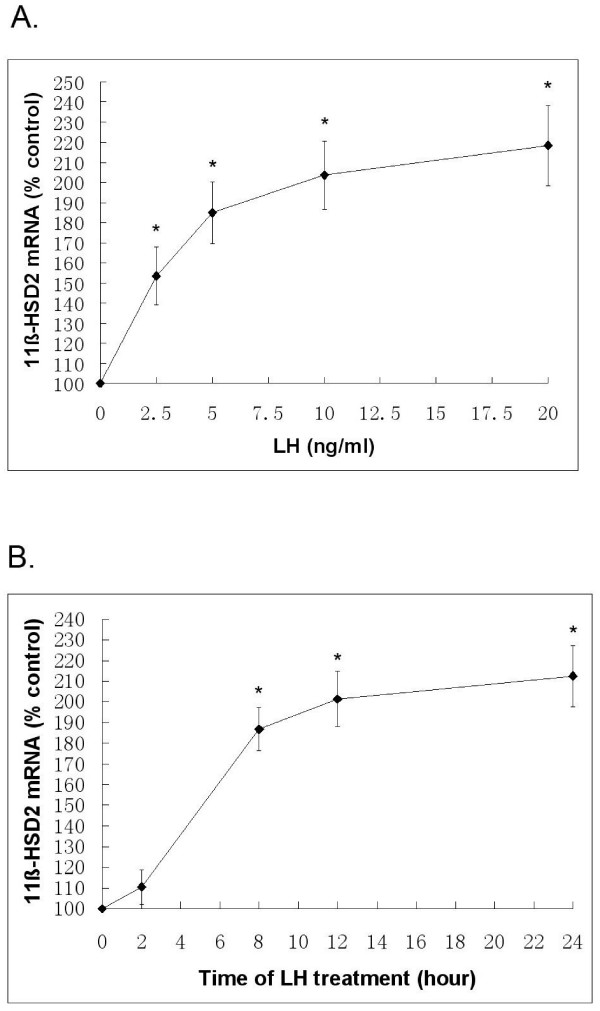
**Effect of LH on expression of mRNA for 11β-HSD2**. Leydig cells were treated with increasing concentrations of LH for 24 h (A). The other cells were treated with LH for 2–24 h (B). At end of treatment, total cellular RNA was isolated, and the steady-state level of 11β-HSD2 mRNA was assessed by relative quantitative RT-PCR, as described in Methods. The expressions of mRNA for 11β-HSD2 in Leydig cells treated with 2.5, 5, 10, or 20 ng/ml LH for 24 h, or 20 ng/mL LH for 8, 12 or 24 h are significantly higher compared to that in intact Leydig cells. Each data point is expressed as percentage of control. *Asterisks *denote significant differences, compared with Control at P < 0.05.

**Figure 2:**
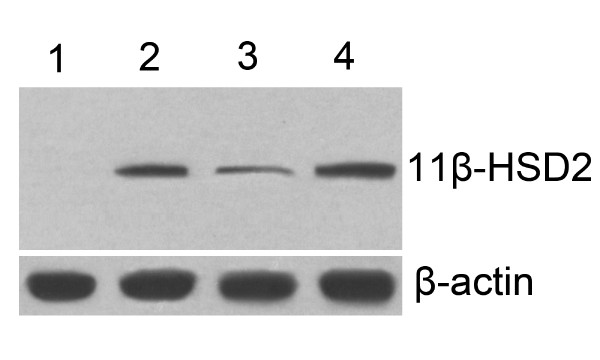
**Effect of LH on expression of 11β-HSD2 protein**. R2C cells were transfected with pcDNA3.0 (lane 1) or pcDNA-hsd11b2 (lane 2); Leydig cells were treated with vehicle (PBS for LH, lane 3) or 20 ng/ml LH for 24 h (lane 4). At end of treatment, levels of 11β-HSD2 protein were determined by western blot analysis as described in Methods.

As shown in Fig. 1A, a concentration-dependent increase in 11β-HSD2 mRNA was found with a maximal effect at 20 ng/ml. Treatment of Leydig cells for 24 h with 20 ng/ml LH led to a significant increase in levels of mRNA. (Fig. 1B). Fig. 2 showed treatment of Leydig cells for 24 h with 20 ng/ml LH increased the level of 11β-HSD2 protein compared with Control.

### Effect of LH on transcription of 11β-HSD2

**Figure 3:**
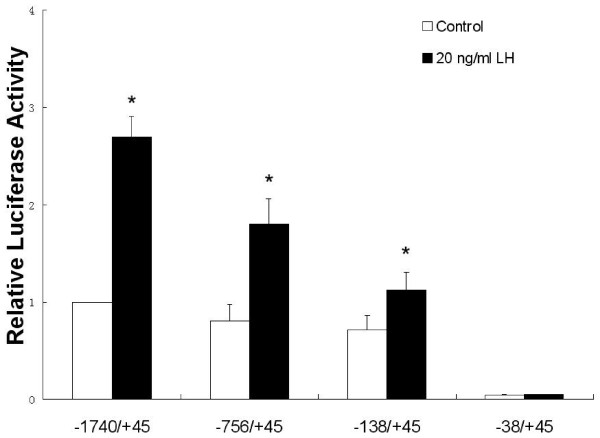
**Effect of LH on 11β-HSD2 promoter activity**. A series of luciferase reporter gene driven by rat 11β-HSD2 promoter region were transfected into mLTC-1 cells. After overnight incubation, the mLTC-1 cells were treated with 20 ng/lm LH for 24 h. The cells were lysed and assayed for luciferase activity. The luciferase activity was compared with the normalized activity of the transfected but not treated cells. These data are representative of at least three independent experiments and are normalized to an internal Renilla control. *Asterisks *indicate differences between groups control versus LH treatment are statistically significant at *P *< 0.05.

To further investigate the effect of LH on the expression of 11β-HSD2, the transcriptional activities of different lengths of rat gene *Hsd11b2 *promoter region were analyzed. LH treatment induced a two-fold increase in the activity of -1740/+45 construct. It was also shown that the -138/+45 region is crucial for the constitutive and LH-induced transactivation of 11β-HSD2 (Fig. 3).

### Effect of LH on 11β-HSD2 enzyme activity

LH significantly increased 11β-HSD2 activity (34.84 ± 2.98%) compared with control cells (15.17 ± 5.22%), *p *<*0.05*.

### Effect of LH on intracellular glucocorticoid concentration

**Figure 4:**
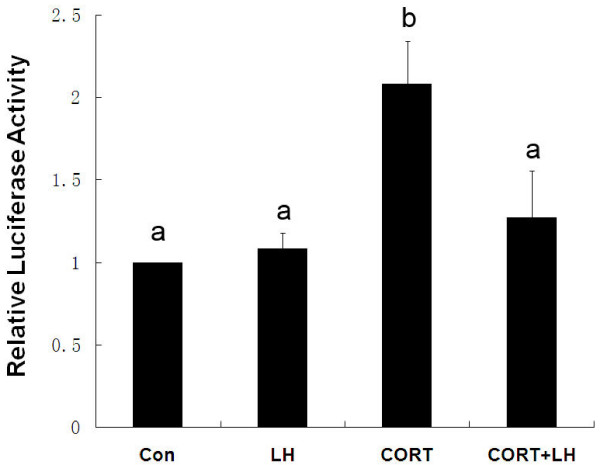
**Effect of LH on intracellular glucocorticoid concentration**. Leydig cells were transfected transiently with a GRE-Luc reporter plasmid, and intracellular glucocorticoid concentration was estimated by GR-mediated transcription of GRE-Luc. Leydig cells were treated with CORT (50 nM), LH (20 ng/mL) or CORT plus LH as described in Methods. Dissimilar superscripts indicate significant differences between groups (*P *< 0.05).

As shown in Fig. 4, 50 nM CORT (within the physiological concentration of CORT in rats) significantly increased the luciferase activity in Leydig cells and LH reduced glucocorticoid-activated transcriptional response of GRE-Luc.

## Discussion

Leydig cells secrete the male sexual hormone testosterone, which is responsible for differentiation of the male phenotype and spermatogenesis in the testes. A direct glucocorticoid receptor (GR)-mediated inhibition of testosterone biosynthesis by excess glucocorticoid has been demonstrated [9]. Some of the solid evidences came from the studies on stress and Cushing's syndrome, in which elevated circulating concentration of glucocorticoid are normally associated with diminished testosterone secretion [1,2]. The recent studies had found that the intracellular level of glucocorticoid is under control by 11β-HSD oxidase activity in Leydig cells [13,15]. Thus far, two distinct forms of 11β-HSD have been identified: 11β-HSD1, which usually is expressed in glucocorticoid target tissue, shows both oxidative and reductive activities and has a relatively low affinity for glucocorticoid substrates [13]; whereas, 11β-HSD2, which usually is expressed in mineralocorticoid target tissue, is an exclusively oxidative isoform with a high affinity for glucocorticoids [14].

Firstly, the presence of 11β-HSD1 in Leydig cell was identified in 1989 [30]. The results of most previous studies demonstrated that 11β-HSD1 is primarily an oxidase and plays a gatekeeper role of oxidatively inactivating glucocorticoids. Lately, the expression of 11β-HSD2 was also detected in rat adult Leydig cells using sensitive real-time PCR and new antibody [15,16]. It is supposed that 11β-HSD2 functions as a protective factor in Leydig cells as well as 11β-HSD1, catalyzing a net oxidative reaction, although its expression is 1000-fold lower than that of 11β-HSD1. One reason is that 11β-HSD2 has a lower Km (15 nM), which is within the range of physiological concentration of glucocorticoid, compared with 11β-HSD1 (Km = 6 μM). Another is that suppression of 11β-HSD2 expression with transfection of antisense oligonucleotides decreased the oxidase activity of Leydig cell by almost 50%.

We had investigated the effect of LH on the expression of 11β-HSD1 at the time when 11β-HSD2 was not identified in Leydig cells [17]. It had been reported that gonadotropic hormone could increase11β-HSD2 expression in Tilapia testis [19], but the relevant mammalian study is not present yet. The results of this present study showed that LH treatment induced expression of 11β-HSD2 mRNA and protein in rat Leydig cells. These results were further confirmed by determining the effect of LH on the transcriptional activity of rat gene *Hsd11b2 *promoter. The luciferase reporter gene assays revealed that 11β-HSD2 promoter-driven transcription increased by more than 100% with LH treatment, consistent with the expression of mRNA and protein. We also found that the -138/+45 fragment, which contains a GC-rich region, is crucial for basic transcriptional activity similar to the results of other studies on human *HSD11B2 *gene [31]. Along with serial deletions of the region between -1740 and -138, the transcriptional activity gradually decreased. This result suggests that several transcription factors may be involved in the LH-regulated expression of 11β-HSD2. This hypothesis needs further investigation in the future.

Actually, some *in vivo *studies had also suggested there is a correlation between the expression of 11β-HSD2 and serum concentration of LH. The study from Ge RS et al. indicated that 11β-HSD2 mRNA and enzyme activity increase continuously and significantly during postnatal development of rat Leydig cells which is accompanied by a gradual increase in serum LH concentration from puberty to maturity [16,32]. Wagner A and Claus R found that changes in expression of 11β-HSD2 appear to parallel with changes in serum concentration of LH in pig Leydig cells after birth [33]. Furthermore, It was shown that GnRH-immunization significantly depresses the plasma concentration of LH as well as 11β-HSD oxidative activity in the boar testis [34]. The data mentioned above and the results of the present study suggest that LH may play an important role in developmental expression of 11β-HSD2 and thereby affecting the intracellular glucocorticoid concentration.

Our previous study revealed that although LH increased the expression of 11β-HSD1 in cultured Leydig cells, the reducase activity of 11β-HSD1 increased slightly and the oxidase activity declined unexpectedly. It has been established that Hexose-6-phosphate dehydrogenase, which generates NADPH through catalyzing glucose-6-phosphate oxidation, is the determinant of the reaction direction of 11β-HSD1 [12]. Moreover, glucose availability may also be involved in determining the reaction direction of 11β-HSD1. Hardy et al. observed that the oxidative and reductive components of 11β-HSD1 activity are unstable *in vitro*. Preparations of freshly isolated Leydig cells display high levels of oxidase activity and lower reductase, but the former declines and the latter rises during incubation *in vitro*. Eliminating the energy sources glucose and pyruvate from the culture medium prevented the changes in oxidoreductase activities, and the 11β-HSD1 oxidase remained predominant in Leydig cells [35]. Similar phenomenon was observed in rat H4IIE hepatoma cells transfected with mouse 11β-HSD1 [36]. Transmembrane glucose uptake mediated by a family of glucose transporters (GLUTs) controls the rate of intracellular glucose availability and metabolism [37]. GLUT8 is highly expressed in rat Leydig cells and positively regulated by hCG which shares the same receptor with LH in Leydig cells [38]. Thus, we supposed that LH-induced increase in glucose availability may be involved in the mechanism for LH-induced change in the enzyme activity of 11β-HSD1. Given that LH reduces the oxidase activity of 11β-HSD1 [17] and increases the expression and enzyme activity of 11β-HSD2 simultaneously, it is unknown what happens to capacity for oxidative inactivation in Leydig cells. To probe the effect of LH on the level of intracellular glucocorticoid receptor activation, the luciferase reporter gene bioassay system was utilized, which had been employed in many studies for the same sake [27,28]. The inducible transcriptional activity of GRE-Luc lies on the extent of the binding of active GR and GRE in the artificial promoter of luciferase gene. As shown in Fig. 4, physiological level of CORT (50 nM) could activate GR signaling pathway, which is consistent with our earlier data that endogenous CORT is capable of inhibiting the steroidogenic capacity of purified rat Leydig cells *in vitro *[3,39], and LH abated the glucocorticoid-induced luciferase activity, i.e. the intracellular concentration of glucocorticoid despite the predominant 11β-HSD1 activity may shift from oxidase to reducase activity hypothetically due to the elevated glucose uptake and NADPH/NADP ratio resulting from treatment with LH [12,38]. Although glucocorticoid exerts adverse effect on testosterone secretion in Leydig cells, some studies suggest that it may be an important factor for Leydig cell development. For example, GR is expressed in Leydig cells at all three stages of pubertal development (mesenchymal-like progenitors, PLC, on day 21, immature Leydig cells, ILC, on day 35, and adult Leydig cells, ALC, on day 90.) and the numbers of dexamethasone-binding sites are higher in ILC and ALC compared with PLC [13]. In controlling the inhibitive effect of glucocorticoid on testosterone secretion during Leydig cell development, LH-induced expression of 11β-HSD2 could play a key role. Interestingly, it was found that glucocorticoid increases the ability of Leydig cell precursors to respond to LH [40]. Therefore, Leydig cells may control intracellular glucocorticoid concentration through a LH-mediated negative feedback mechanism during development.

## Conclusion

In summary, LH increases the expression and enzyme activity of 11beta-HSD2, and therefore enhances capacity for oxidative inactivation of glucocorticoid in rat Leydig cells *in vitro*. Thus, this study together with other published data has revealed an integrated relationship between LH and glucocorticoid in Leydig cells, which may help us deepen our understanding of the intricate mechanism for regulation of testosterone secretion in Leydig cells.

## Competing interests

The authors declare that they have no competing interests.

## Authors' contributions

HBG conceived and designed the study. QW performed Real-Time PCR, Western Blot, 11beta-HSD2 enzyme activity assay, and drafted the manuscript. PZ performed construction of vectors, Luciferase activity assays, and data analysis. All authors read and approved the final manuscript.
